# 

**Published:** 1893-08

**Authors:** 


### NOTICES.

### Southern Dental Association.

DECATUR, ALA., JULY 14TH, 1893.

*My Dear Doctor:*

The Committee of Arrangements have secured the Kindergarten Hall, No. 10 Van Buren street, Chicago, Ill., as a place for holding the business meeting of the Southern Dental Association during the World's Columbian Dental Congress. Time for the meeting will be Friday, August 11th, at 10 a. m.; this being the nearest date we could secure previous to the meeting of the Congress, which is on the following Monday, August 14th.

It is very important that every member of the Southern Dental Association should be present, as it will add not only to the success of the meeting and the great personal benefit to be derived from it, but it will uphold the importance of the Southern in this great Congress which has representatives from all over the world.

B. HOLLY SMITH, President.
S. W. FOSTER, Secretary.

### National Association of Dental Examiners.

The twelfth annual meeting of the National Association of Dental Examiners will be held in the house of the Columbian Dental Club, No. 300 Michigan avenue, Chicago, Friday, August 11th, at 10 o'clock a. m. Attention is called to the following resolution passed August 5th, 1891:

*Resolved*: That the various State Boards of Dental Examiners be requested, each year in season for the annual meeting, to forward to the secretary a written report of their examinations, accompanied by a detailed or tabulated statement.

W. E. MACGILL, Secretary.

### American Dental Association.

The Thirty-third Annual Session of the American Dental Association will be held in Kindergarten College Hall, 10 Van Buren street, Chicago, commencing Saturday, August 12th, 1893, at 10 o'clock A. M.

GEO. H. CUSHING,
*Recording Secretary.*

### CONTENTS.

COMMUNICATIONS.

A MONTHLY MAGAZINE DEVOTED TO THE INTERESTS OF THE

### DENTAL PROFESSION.

*Terms Reduced to* $2.00 *Per Year. Single Copies,* 25 *Cents.*

### EDITED BY PROF. J. TAFT, D.D.S.

—AND—

### Published by SAM'L A. CROCKER & CO., Ohio Dental and Surgical Depot.

The volume commences in January and closes in December.

The REGISTER being issued monthly is always fresh, therefore Indispensable to the practitioner who desires to keep posted up with the new inventions and improvements which are daily developed. Neither expense nor effort will be spared to give value and variety to its contents. Articles will be illustrated with well executed engravings whenever they will add to the interest or assist to explanation.

Its extensive circulation and frequency of issue make it one of the BEST DENTAL ADVERTISING MEDIUMS in the United States.

All subscriptions must begin with January or July.

Local or special notices, five lines or less, will be inserted for $3 each insertion ; over five lines, 50 cts. per line.

All advertising bills payable in advance, or at furthest, after the issue of the first number containing the advertisement. All transient advertisements to be paid for in advance.

☞ CONTRIBUTIONS SOLICITED.

Exclusively Editorial Communications should be addressed to the Editor.

The latest number of the REGISTER may be ordered with or without other goods at any time, and all letters should be addressed to

